# *EZH2* and *CD79B* mutational status over time in B-cell non-Hodgkin lymphomas detected by high-throughput sequencing using minimal samples

**DOI:** 10.1002/cncy.21262

**Published:** 2013-01-29

**Authors:** Mauro Ajaj Saieg, William R Geddie, Scott L Boerner, Denis Bailey, Michael Crump, Gilda da Cunha Santos

**Affiliations:** 1 Department of Laboratory Medicine and Pathobiology, University of TorontoOntario, Canada; 2 Laboratory Medicine Program, University Health NetworkToronto, Ontario, Canada; 3 Division of Medical Oncology and Hematology, University Health NetworkToronto, Ontario, Canada

**Keywords:** B-cell non-Hodgkin lymphoma, fine-needle biopsy, DNA mutational analysis, *EZH2*, *CD79B*, MassARRAY spectrometry

## Abstract

**BACKGROUND:** Numerous genomic abnormalities in B-cell non-Hodgkin lymphomas (NHLs) have been revealed by novel high-throughput technologies, including recurrent mutations in *EZH2* (enhancer of zeste homolog 2) and *CD79B* (B cell antigen receptor complex-associated protein beta chain) genes. This study sought to determine the evolution of the mutational status of *EZH2* and *CD79B* over time in different samples from the same patient in a cohort of B-cell NHLs, through use of a customized multiplex mutation assay.

**METHODS:** DNA that was extracted from cytological material stored on FTA cards as well as from additional specimens, including archived frozen and formalin-fixed histological specimens, archived stained smears, and cytospin preparations, were submitted to a multiplex mutation assay specifically designed for the detection of point mutations involving *EZH2* and *CD79B*, using MassARRAY spectrometry followed by Sanger sequencing.

**RESULTS:** All 121 samples from 80 B-cell NHL cases were successfully analyzed. Mutations in *EZH2* (Y646) and *CD79B* (Y196) were detected in 13.2% and 8% of the samples, respectively, almost exclusively in follicular lymphomas and diffuse large B-cell lymphomas. In one-third of the positive cases, a wild type was detected in a different sample from the same patient during follow-up.

**CONCLUSIONS:** Testing multiple minimal tissue samples using a high-throughput multiplex platform exponentially increases tissue availability for molecular analysis and might facilitate future studies of tumor progression and the related molecular events. Mutational status of *EZH2* and *CD79B* may vary in B-cell NHL samples over time and support the concept that individualized therapy should be based on molecular findings at the time of treatment, rather than on results obtained from previous specimens. Cancer (Cancer Cytopathol) 2013;121:377–386. © 2013 American Cancer Society.

Significant advances in the understanding of the molecular complexity and alterations involved in the pathogenesis and progression of B-cell non-Hodgkin lymphomas (NHL) have been achieved with the emergence of high-throughput molecular technologies such as array comparative genome hybridization and next generation sequencing. Extensive research based on these novel technologies using material from surgical specimens (paraffin-embedded tissue and frozen specimens) and cell lines has unveiled a large number of chromosomal abnormalities, as well as somatic mutations of potential therapeutic importance.^1^–^4^

Recurrent point mutations involving the *EZH2* and *CD79B* genes have been reported in follicular lymphoma, diffuse large B-cell lymphoma (DLBCL) and not in marginal zone lymphoma.^5^–^7^ *EZH2* (enhancer of zeste homolog 2) represents 1 of 3 subunits of polycomb-repressive complex 2 (PRC2) and is responsible for catalyzing the methylation of PRC2’s target, lysine-27 of histone 3 (H3K27), an epigenetic marker associated with transcriptional silencing and involvement in cellular differentiation, morphogenesis, and organogenesis.^8^–^11^ Mutations in the Tyr641 residue in the SET (Su[var#x005D;3-9, Enhancer-of-zeste, Trithorax) domain of *EZH2* have shown to act in a dominant fashion increasing H3K27 tri-methylation, conferring a gain of function and involvement in carcinogenesis.^12^ For *CD79B*, point mutations causing the substitution of the first tyrosine of the ITAM (immunoreceptor tyrosine-based activation) motif (Y196) cause the B-cell receptor to form clusters in the cytoplasm, leading to the so-called “chronic active B-cell receptor signaling,” with subsequent constitutive downstream activation of the nuclear factor-κB pathway.^5^–^13^ These 2 mutations therefore have a major role in lymphomagenesis and stand out as putative candidates for molecular targeted therapies in the future.^12^–^14^

There is a recurrent concern over which is the most appropriate sample to be used for genomic analysis, based on the fact that changes in the molecular status may occur in malignant neoplasms between initial diagnosis and relapse, with the acquisition of novel abnormalities. In breast cancer, for example, there is mounting evidence that recurrent or metastatic tumors should be re-biopsied, due to differences seen in molecular profile and discrepancies in receptor status between primary and recurrent samples.^15^ Recently, differences in mutational status were found between paired ductal carcinomas in situ and the adjacent invasive component in breast cancer.^16^ Different chromosomal abnormalities and copy number alterations were also seen in follicular lymphomas and the transformed DLBCL samples^17^ and in primary and metastatic samples from lung and colon cancer.^18^–^19^

Recent reports describe the use of cytology specimens as a source of molecular information, with reliable results using novel technologies and DNA extracted from archived cytological preparations and fresh samples.^20^–^22^

In this study, we sought to detect mutations in patients with B-cell NHL and compare mutational status over time using different cytological and histological preparations as the substrate for a customized multiplex mutation assay for the detection of point mutations involving *EZH2* and *CD79B* using the MassARRAY spectrometry platform.

## MATERIALS AND METHODS

### Sample Collection

Residual material remaining after following a previously detailed protocol^23^ for manipulation of cytological specimens from patients who had a diagnosis of lymphoma was prospectively collected from July 2010 to October 2011. Briefly, as part of the diagnostic workup, assessment of the adequacy and triage of the samples was performed on-site for all cases at the time of the procedure. Part of the material was smeared onto slides for immediate assessment and the remaining material in the syringe was rinsed in sterile saline and triaged for ancillary studies. Fresh cell suspensions from the needle rinse were then used for immunophenotyping by laser scanning cytometry or flow cytometry and for preparation of cytospins, which are subsequently used for assessment of the proliferation index by MIB-1, other immunohistochemistry analyses, and cytogenetic studies by fluorescence in situ hybridization (FISH). Information on the monoclonal antibody clones and concentrations used, as well as fluorochromes, FISH probes, and manufacturers have been previously detailed.^24^–^25^ Whenever possible, a concurrent core biopsy is also collected at the time of the procedure.^23^

After the diagnostic workup was deemed complete, residual material from the needle rinse cell suspension was collected on the FTA cards (Whatman, Kent, UK) as described.^21^ The cards were allowed to air dry for at least 24 hours before being stored in a plastic bag at room temperature for a variable number of days until DNA extraction. Material from 5 cases of confirmed benign lymphoid hyperplasia was also collected on FTA cards and used as negative controls.

In order to compare the molecular alterations found in the cases collected in the FTA cards to other specimens from the same patient, an electronic search was conducted to retrospectively retrieve all additional samples with a diagnosis of B-cell NHL. All previous, concurrent, and subsequent samples with available cytological or histological material were retrieved from the archival files and tissue biobank and used for DNA extraction. Samples consisted of archived Romanowsky-stained smears, unstained archived cytospin preparations, formalin-fixed paraffin-embedded (FFPE) tissue and frozen tissue specimens. Archived unstained cytospins had been stored at −20 °C, and frozen surgical specimens had been stored at −70 °C for a variable number of years at the time of collection.

Clinicopathological data retrieved from the patients’ electronic reports included age, sex, location, date of the sample collection, results of immunophenotyping, immunohistochemistry including MIB-1 proliferation index and FISH studies, as well as final cytological and histological diagnostic interpretations. This study was approved by the University Health Network Research Ethics Board.

### DNA Extraction

DNA from the FTA cards was extracted from two 3-mm punches of the card, following the manufacturer’s protocol for Classic cards, as previously detailed.^21^ DNA from the additional specimens was extracted according to previously published protocols, with minor alterations.^20^–^26^ As an initial preparation for DNA extraction, Romanowsky-stained smears were left in xylene for cover-slip removal for a variable number of days dependent on their age, and subsequently soaked in 100% ethanol to remove the xylene. For the FFPE surgical specimens, five 10 μm unstained sections placed on charged slides were retrieved from the blocks. For the frozen specimens, ten 10 μm sections were obtained from the material stored at −70 °C. The first and last sections were stained with hematoxylin and eosin for adequacy control, and the remaining 8 sections were placed in 2 charged slides and used for the extraction. After this initial step, 20 μL of animal tissue lysis buffer (buffer ATL; Qiagen, Valencia, Calif) was placed on the slides and material was scraped with a sterile blade and transferred to a collection tube. Deparaffinization was then performed for the FFPE tissue with xylene followed by ethanol 75%, and removal of the embedding medium used for the frozen specimens (Tissue Tek optimal cutting temperature #x005B;OCT#x005D; compound; Sakura Finetek, Alphen aan den Rijn, Netherlands) was done using a high TE buffer solution. The scraped material underwent tissue lysis at 60 °C overnight with 100 μL of a 4:1 solution of buffer ATL and proteinase K, and subsequent DNA isolation using a commercially available spin-column protocol (DNeasy Blood and Tissue Kit; Qiagen, Valencia, Calif) for the smears, cytospins and frozen specimens and a manual standard phenol-chloroform method for the FFPE tissue.

Nucleic acid extracts were stored at −70 °C until use for molecular analysis. For all cases, DNA concentration was assessed by NanoDrop 1000 spectrophotometer (version 3.7.1; ThermoFisher Scientific, Wilmington, Del).

### MassARRAY Spectrometry

All cases were submitted to high-throughput multiplex mutation profiling using the MassARRAY platform (Sequenom, San Diego, Calif). Four assays were developed in the same panel for the detection of nucleotide changes in codon 196 of *CD79B* and codon 641 of *EZH2*. These 2 codons have the same nucleotide sequence (TAC), and point mutations have been reported in their first and second positions. Because of the multiplexed nature of this type of assay, multiple mutations can be detected simultaneously using 1 panel. SNP genotyping multiplex reactions were designed using Sequenom’s assay design tools. In each of the 4 assays, 10 ng of DNA was used. Polymerase chain reaction (PCR) was carried out on a 384-well plate with specifically designed primers flanking the regions of interest and extension primers that bind adjacent to the mutation sites. Extension primers used were the following: 5′-GAAAAATGAATTCATCTCAGAA-3′ and 5′-GCCTTACCTCTCCACAG-3′ for the first and second positions of *EZH2* and 5′-GGAGGAAGATCACACC-3′ and 5′-CCCTCTCCTTACCTCG-3′ for the first and second positions of *CD79B*, respectively. Unincorporated deoxynucleotide triphosphates (dNTPs) were dephosphorylated by treatment with shrimp alkaline phosphatase (SAP), and the products were transferred onto a 384-well SpectroCHIP bioarray (Sequenom) using the Sequenom RS-1000 MassARRAY Nanodispenser and analyzed using the Sequenom MassARRAY Analyzer Compact. Assessment of the performance of the assays and clustering of genotypes was done using the Typer 4.0 software (Sequenom). Successful assays were defined as yielding definitive results (either positive or negative for mutation for each of the 4 assays) for the samples tested.

Several blank (water) samples were included with each plate to ensure that no DNA contamination and nonspecific primer binding were present. Visual inspection of the spectra was performed for each assay and for any samples with low spectra quality (those with low peak signals). Manual inspection on all assays was conducted to confirm the analysis provided by the MassARRAY Typer Analyzer software 4.0 (Sequenom) on the genotype calls, to identify actual mutant peaks from salt peaks or nonspecific background peaks.

### Sanger Sequencing

PCR followed by direct sequencing was performed for confirmation of all cases found positive for *EZH2* or *CD79B* mutations by MassARRAY spectometry and was also for all the cases in which the assay failed to yield results. For each reaction, 5 μL of the eluate was used. For *EZH2*, the primers were used flanking the region containing the tyrosine domain in exon 15 (forward, 5′-TTTGTCCCCAGTCCATTTTC-3′; reverse, 5′-AAGGCAGTTTATGGCAATTCA-3′), that produces a 278–base pair amplicon. For *CD79B*, the primers used were (forward 5′-TCTTGCAGAATGCACCTCAC-3′, reverse 5′-CAGGCCCTGGAGACATTAAG-3′), producing a 280–base pair amplicon. Each PCR reaction was subjected to 95 °C for 15 minutes, followed by 35 cycles, with denaturing at 94 °C for 30 seconds, annealing at 56 °C for 30 seconds, and extension at 72 °C for 45 seconds with 5 minutes incubation at 72 °C followed by 4 °C. PCR products were analyzed by electrophoresis on 2% agarose gels. Unincorporated primers and dNTPs were removed from PCR using 0.4 U of Exonuclease I (USB) and 0.2 U of SAP and incubated at 37 °C for 40 minutes, followed by inactivation of the enzymes at 85 °C for 5 minutes. Bidirectional direct sequencing of the PCR products was performed using the Applied Biosystems BigDye Terminator Cycle Sequencing Kit, version 3.1 and the ABI-Prism 3130xl Genetic Analyzer (Applied Biosystems, Carlsbad, Calif). Sequence data were analyzed using the Codon Code Aligner software (Codon Code, Dedham, Mass) followed by manual review of all cases.

## RESULTS

### Patient Characteristics

A total of 80 non-Hodgkin B-cell lymphomas from 75 patients were collected in the FTA cards. There were 40 males and 35 females, with a median age of 63 years old (range, 37-91). Clinicopathological data for the cases is summarized in Table1. The median number of days the material was stored in the cards until DNA extraction was 32 (range, 1-223). Cell concentrations from samples varied from 1.4 × 10^6^ to 200 × 10^6^ cells/mL, with an average of 19.3 × 10^6^ cells/mL. FNA samples were obtained from lymph nodes (69) and extranodal sites (11), as follows: lung (3), mediastinum (2), retroperitoneum (2), parotid (2), and subcutaneous (2).

**Table 1:** List of Non-Hodgkin B-Cell Lymphomas Stored on FTA Cards

Final Cytological Diagnosis	No. of Cases (Patients)	Age^a^ (Range)	Sex (M, F)	IP^b^	MIB-1, % (Range)	FISH^b^	Concurrent Surgical^b^
**Cases subtyped**							
DLBCL	14 (12)	59.5 (42-90)	7 M, 5 F	13	70.6 (40-98)	10	5
FL	32 (31)	60 (38-87)	13 M, 18 F	32	14.2 (3-40)	18	13
MCL	5 (5)	69 (48-77)	4 M, 1 F	5	19.25 (2-45)	2	1
MZL	6 (6)	63 (50-74)	3 M, 3 F	6	8 (4-10)	6	2
SLL	6 (6)	72.5 (37-86)	3 M, 3 F	6	11.6 (5-20)	5	2
LPL	2 (2)	70.5 (64-77)	1 M, 1 F	2	10^c^	1	N/A
U-DLBCL/BL (DHL)	2 (2)	67 (63-71)	2 F	2	85 (80-90)	2	2^d^
**Cases not subyped**							
BNHL	3 (2)	67 (65-69)	2 M	2	N/A	N/A	1
LBCL	6 (6)	75.5 (55-91)	5 M, 1 F	6	60.2 (40-95)	4	3
SBNHL	4 (3)	64 (55-80)	2 M, 1 F	3	3.5 (2-5)	3	1

a Presented in years, median.

b Number of events for each diagnosis listed.

c Data for only 1 case.

d One case had a discordant surgical diagnosis.

Abbreviations: BNHL, B-cell non-Hodgkin lymphoma; DLBCL, diffuse large B-cell lymphoma; FISH, fluorescence in situ hybridization; FL, follicular lymphoma; IP, immunophenotyping; LBCL, large B-cell lymphoma; LPL, lymphoplasmacitic lymphoma; MCL, mantle cell lymphoma; MZL, marginal zone lymphoma; N/A, not available; SLL, small lymphocytic lymphoma; SBNHL, small B-cell non-Hodgkin lymphoma; U-DLBCL/BL (DHL), unclassifiable, with features intermediate between diffuse large B-cell lymphoma and Burkitt lymphoma (dual-hit lymphoma).

### B-Cell NHL Subclassification

Subtyping according to the latest World Health Organization classification was possible for 67 cases (83.7%) and the final cytological diagnoses were as follows: 32 follicular lymphoma (FL), 14 DLBCL, 6 small lymphocytic lymphomas (SLL), 6 marginal zone/MALT (extranodal marginal zone lymphoma of mucosa-associated lymphatic tissue) lymphomas, 5 mantle cell lymphomas (MCL), 2 lymphoplasmacytic lymphomas (LPP), and 2 “unclassifiable with features intermediate between DLBCL and Burkitt lymphoma (BL)” (U-DLBCL/BL). Thirteen cases (16.3%) could not be fully subtyped and had a final cytological diagnosis of B-cell NHL (3), large B-cell lymphoma (6), and small B-cell NHL (4). Immunophenotyping using laser scanning cytometry was successfully performed for all patients. MIB-1 proliferation index was assessed in 61 (76.2%) cases. FISH studies were performed for 51 (63.7%) cases. Gene rearrangements were found in 30 cases and included translocations for *IGH/BCL-2* (20 cases) and *IGH/CCND1* (2 cases) as well as rearrangements for *MYC* (1 case) and *MALT-1* (2 cases). Three cases had both *IGH/BCL-2* translocation and *MYC* rearrangement. Other chromosomal abnormalities were seen in 2 cases. In 19 cases, no cytogenetic alterations were found. The results from FISH were inconclusive in one case, and the assay failed in another case.

The final cytological diagnoses were compared to the final histological diagnoses of the corresponding surgical specimens obtained at the time of the procedure. A concurrent surgical biopsy was available for 30 cases and agreement between the final cytological and histological diagnoses was achieved in 29 cases (96.6%). The only discordant case had a final cytological diagnosis of U-DLBCL/BL and final histological diagnosis of lymphoblastic lymphoma/leukemia.

### Additional Samples From the Cases Collected on the FTA Cards

**Figure 1:**
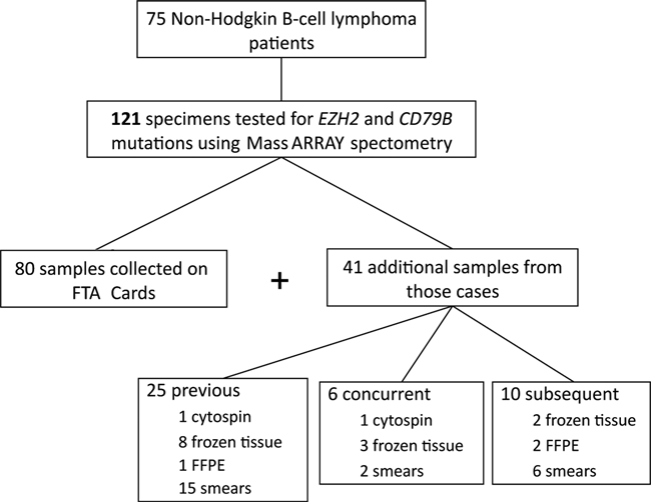
Flow chart shows the total number of samples and patients enrolled in the study, as well as the types of specimens used and their connection to the samples collected on the FTA cards.

In 26 patients, multiple samples were collected over time and were used for comparison of mutation status. A total of 41 samples with available material for DNA extraction were collected from the department archives and tissue biobank. They corresponded to 25 previous, 6 concurrent, and 10 subsequent samples (Fig. 1). Type of specimens collected included 23 archived stained smears, 13 frozen surgical specimens, 2 archived cytospins, and 3 FFPE samples. Diagnoses included 28 FL, 5 DLBCL, 2 cases of combined DLBCL/FL, 2 SLL, 2 small B-cell NHL, 1 marginal zone lymphoma, and 1 FL in situ.

### DNA Extraction

DNA was successfully extracted from all specimens. The mean DNA concentration extracted from each type of sample is shown in Table2. DNA concentration from the samples collected on FTA cards varied from 0.89 to 21.89 ng/μL, with an average of 6.44 ng/μL. For the additional samples, mean DNA concentrations (and ranges) were as follows: 23.32 ng/μL (0.89-21.89 ng/μL) for frozen tissue specimens, 10.1 ng/μL (5.6-14.7 ng/μL) for FFPE tissue, 6.69 ng/μL (0.38-26.87 ng/μL) for archived smears, and 4.75 ng/μL (3.2-6.3 ng/μL) for the cytospin slides.

**Table 2:** Total DNA Yield and Successful Rate of Analysis of Different Types of Samples

Type of Sample	N^a^	DNA Mean Concentration, ng/µL (Range)	% of Successful Results Using MassARRAY Spectometry^b^
FTA card	80	6.44 (0.89-21.89)	99.7
Frozen tissue	13	23.32 (2.7-115.6)	100
FFPE tissue	3	10.1 (5.6-14.7)	91.6
Archived smears	23	6.69 (0.38-26.87)	65.2
Archived cytospins	2	4.75 (3.2-6.3)	75

a Total number of specimens used.

b Based on the total number of assays (n × 4).

Abbreviation: FFPE, formalin-fixed, paraffin-embedded.

### MassARRAY Spectometry Analysis Followed by Sanger Sequencing

High-throughput mutation analysis using MassARRAY spectrometry was performed in 121 samples from 80 patients. A total of 4 assays (2 for the Y646 *EZH2* mutation and 2 for the Y196 *CD79B* mutation) were performed for each sample, totaling 484 assays. The success rate of these analyses for each type of specimen is shown in Table2. Frozen specimens had a 100% success rate, followed by FTA cards (99.7%), FFPE (91.6%), cytospins (75%), and archived smears (65.2%). All cases of lymphoid hyperplasia used as negative controls were successfully analyzed and no mutations were found. In addition, corresponding surgical (FFPE/frozen) specimens were also tested as gold standard internal quality control for the results obtained by cytological samples. Core biopsies or lymph node excisional specimens (frozen/FFPE tissue) were available for testing for 12 patients, and results mirrored the mutational status of the cytological samples in all of the cases (Table3).

**Table 3:** Cases With Validation of the Results Using Correspondent Surgical Specimens

Case	Type of Specimen	Mutation Result	Correspondent Frozen/FFPE
10	FTA	Neg	Neg^b^
12.1	Smear	*EZH2* Y646F	*EZH2* Y646F
48	FTA	*EZH2* Y646C	*EZH2* Y646C^b^
50.2	Smear	Neg	Neg
51^a^	FTA	Neg	Neg
55	FTA	*CD79B* Y196C	*CD79B* Y196C^b^
62.1	Smear	Neg	Neg
66	FTA	Neg	Neg
68	FTA	Neg	Neg
70	FTA	*EZH2* Y646N	*EZH2* Y646N
77	FTA	Neg	Neg
81	FTA	Neg	Neg

a There are 2 previous frozen samples for this case.

b These cases have both frozen and FFPE tissue specimens, with the same results.

Abbreviations: FFPE, formalin-fixed paraffin-embedded; Neg, negative.

Direct sequencing using reverse and forward primers for each gene tested was subsequently applied for all samples that had failed MassARRAY spectrometry assays. All of these specimens were successfully analyzed by Sanger sequencing, and a definitive mutational status was therefore available for all samples. In one sample (76.1), a mutation in *EZH2* was found solely by direct sequencing. In order to confirm the results obtained by spectrometry, all the samples positive for mutation by MassARRAY analysis were also Sanger sequenced. The mutations were confirmed for all samples but one (29.2). This sample was found to be positive for an *EZH2* mutation (Y646S) by MassARRAY analysis, but direct sequencing could not confirm the presence of the mutation and was therefore excluded from the analysis of the positive cases. The specimen corresponded to a previous smear from a FL case collected on the FTA card, which was negative for the presence of any mutation by MassARRAY. Overall, considering all the assays with positive results, confirmation was possible in 71 of 72 (98.6%) of the samples.

### *EZH2* and *CD79B* Mutations and B-Cell NHL subtypes

All the samples with positive results for mutations involving *EZH2* or *CD79B* found by MassARRAY spectrometry and confirmed by Sanger sequencing are shown in Table4. In total, 18 samples (14.9%) showed heterozygous mutations for either of the genes.

**Table 4:** Positive Cases (FTA card) for *EZH2* and *CD79B* Mutations, With List of Additional Samples Tested

Case	Type of Specimen	Final Diagnosis	Relation	Location	Mutation	Direct Sequencing^a^
***EZH2***						
1	FTA card	DLBCL		LN right iliac	Y646S	C
1.1	Smear	FL I-II	Previous	Retroperitoneal mass	Y646S	C
3^b^	FTA card	U-DLBCL/BL (DHL)		LN left neck supraclavicular	Y646S	C
12	FTA card	FL I-II		LN right supraclavicular	Neg	NT
12.1	Smear	FL III	Previous	LN left inguinal	Y646F	C
12.3	Frozen	DLBCL (60%)/FL IIIA-IIIB (40%)	Previous	LN left inguinal	Y646F	C
21^b^	FTA card	FL I-II		LN left neck	Y646S	C
25	FTA card	DLBCL		Retroperitoneal mass	Neg	NT
25.2	Smear	FL I-II	Previous	LN right inguinal	Y646F	C
27^b^	FTA card	DLBCL		Intra-abdominal mass	Y646F	C
48	FTA card	LBCL		LN left upper neck	Y646C	C
48.1	Frozen/FFPE	FL IIIA	Subsequent	LN left cervical	Y646C	C
58^b^	FTA card	LBCL		LN mesenteric	Y646F	C
62	FTA card	FL I-II		LN left supraclavicular	Neg	NT
62.1	Smear	FL I	Previous	LN right axila	Neg	NT
62.2	Frozen	FL II	Previous	LN right axila	Neg	NT
62.3	Smear	FL	Previous	LN right axila	Neg	NT
62.5	Smear	FL I	Previous	LN right supraclavicular	Neg	NT
62.6	Smear	FL I-II	Concurrent	LN left neck level II	Y646F	C
70	FTA card	FL I-II		LN left supraclavicular	Y646N	C
70.1	Frozen	FL II-IIIA	Previous	LN right supraclavicular	Y646N	C
76	FTA card	FL I-II		LN right posterior cervical	Y646N	C
76.1	Smear	Small B-Cell NHL	Previous	LN right submandibular	Y646N	C
***CD79B***						
55	FTA card	LBCL		LN right posterior neck	Y196C	C
55.1	Frozen/FFPE	DLBCL	Subsequent	LN right posterior neck	Y196C	C

a Confirmation done by direct sequencing.

b No additional samples were available for DNA extraction for these cases.

Abbreviations: C, confirmed; DLBCL, diffuse large B-cell lymphoma; FFPE, formalin-fixed paraffin-embedded; FL, follicular lymphoma; LBCL, large B-cell lymphoma; LN, lymph node; NHL, non-Hodgkin lymphoma; NT, not tested; U-DLBCL/BL (DHL), unclassifiable, with features between diffuse large B-cell lymphoma and Burkitt lymphoma (dual hit lymphoma).

Sixteen samples (13.2%) from 11 cases presented with the Y646 mutation for *EZH2*. Diagnoses included 9 FL, 2 DLBCL, 2 LBCL, 1 U-DLBCL/BL, 1 composite DLBCL/FL, and 1 small B-cell NHL. This last sample (76.1) could not be properly subtyped at the time of diagnosis due to insufficient number of tumor cells, but a subsequent sample tested from the same case (76) had a diagnosis of FL, also positive for the same *EZH2* mutation. Four variations of the mutation were seen: 6 samples showing Y646F substitution, 4 with Y646S substitution, 4 with Y646N substitution, and 2 with Y646C substitution. No different nucleotide substitutions were seen within samples from the same case. When specifically analyzing FL and DLBCL/LBCL, lymphomas for which these mutations were previously described, *EZH2* mutations were seen in 9 of 60 FL tested (15%) and in 4 of the 25 DLBCL/LBCL samples tested (16%).

Two samples (1.6%) from one case showed the Y196 mutation for *CD79B*. Diagnoses were LBCL and DLBCL. They both showed a Y196C substitution. When considering only the DLBCL/LBCL tested, mutation rate was 8%. No case showed concomitant *EZH2* and *CD79B* mutations.

### Comparison of the *EZH2* and *CD79B* Mutations Over Time

**Figure 2:**
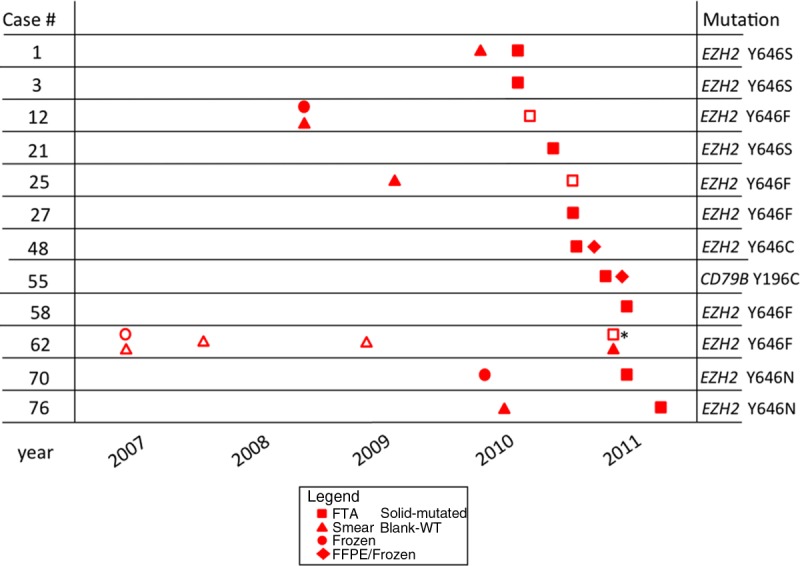
Schematic representation shows the positive cases for the *EZH2* and *CD79B* mutations and the evolution of their mutational status over time. The sample marked with an asterisk is from a different site than its concurrent smear.

Seven cases presenting with *EZH2* mutations had additional samples for comparison. In 4 patients, both previous and current samples were positive for mutation: 1 case of a previous FL with a subsequent DLBCL, 1 case of a previous FL with subsequent LBCL, 1 case of FL recurring as FL, and 1 case of small B-cell NHL recurring as FL. For 2 patients, the previous samples were positive for mutation and the current samples were negative: 1 case with 2 previous samples (a combined DLBCL/FL and a FL) which recurred as FL and another case of FL with a subsequent DLBCL. In the remaining case, 4 previous samples and the current sample (FTA), all with diagnoses of FL, were negative for mutation, but a concurrent cytological sample (smear) from a different location to the sample collected on an FTA card also diagnosed as FL was positive for *EZH2* mutation. For the case presenting with *CD79B* mutation, there were 2 positive samples: a current LBCL and a subsequent DLBCL. Comparison of the mutation status is illustrated in Fig. 2.

## DISCUSSION

We have demonstrated that the mutational status of B-cell NHL samples from the same patients collected at distinct times during the course of their disease varied in one-third of the positive cases. Successful and reliable results were obtained using minimal residual fresh unfixed material from FNAs stored in FTA cards as well as from archived smears and cytospin preparations. In addition, with the application of a customized multiplex mutation assay, we found mutations involving *EZH2* and *CD79B* in cases of FL and DLBCL in a similar rate to that reported for surgical pathology specimens.

Comparison was possible for multiple samples from the same patient, with evaluation of the mutation status at different time points. Two previous reports on the analysis of *EZH2* Y646 mutations in B-cell NHL have compared initial and recurrent samples from the same patients.^6^–^27^ In both studies, however, only histological specimens were used, and comparisons were available for only 2 specimens from the same case. In one of the studies, although there seemed to be a tendency for an increased frequency of *EZH2* mutation in the cases with transformed samples, no association between *EZH2* mutation and FL transformation was seen.^27^ These results are in agreement with our study, where *EZH2* mutational status varied over the course of the disease within specimens from the same patient.

Discrepant mutational status of *EZH2* over time seen in our series could be explained by genetic heterogeneity.^28^ The case with previous wild-type tumors and a subsequent mutated sample may have acquired genetic abnormalities over time, a common phenomenon in lymphomas.^17^,^29^ For the 2 cases in which the mutation was only seen in the initial samples, discrepancies may be explained wherein samples may not be truly “recurrent,” but a de novo neoplasm unrelated to the primary samples and therefore exhibiting different mutational statuses.^31^ Other possibilities are tumor heterogeneity or parallel clonal evolution of the initial and recurrent tumors. In many instances, primary and sequential transformed biopsies of FL did not share all abnormalities, indicating that rather than evolving from the initial sample, the sequential biopsies could have often come from a common precursor, with parallel clonal evolution, and one of the “subclones” may have given rise to large-cell lymphomas.^32^ Our results support the idea of a shift from the analysis of the primary tumor alone, to a combination of primary tumor, circulating tumor cells, metastatic deposits, and cell-free DNA to capture the full extent of the genetic heterogeneity present.^28^ The successful use of all types of specimens, including limited resources such as archived smears and cytospin preparations, can further be explored to elucidate the discrepancies seen over time in a mutation-enriched cohort of patients.

Mutations at the tyrosine in the SET domain of *EZH2* were seen in our patients at a similar rate to previous reports.^27^,^31^,^33^,^34^ For *CD79B* mutations, our study showed a lower rate, being observed in just 1 case.^5^ Variations of the mutations reported here for either *EZH2* or *CD79B* were the same as previously described, and no new variants were seen. Of interest, all cases with 2 mutated samples showed the same type of nucleotide substitution. Our study and previous reports have demonstrated that tumor specimens are heterozygous for the Y646 *EZH2* mutations.

The use of MassARRAY spectrometry combined with Sanger sequencing provided definitive results for all types of samples tested in our study, although some of the biospecimens were not initially collected for the purpose of performing molecular analyses. Only one case found to be positive for the *EZH2* mutation using MassARRAY spectrometry was not confirmed by direct sequencing. A possible explanation for this discrepancy might be that a small percentage of tumor cells are present in this sample and may have fallen into the range of 5% to 10% of mutated tumor cells that could be detected by MassARRAY spectrometry but would not be detectable by direct sequencing.^35^–^36^ Alternatively, it might also represent a false positive case, a less probable situation because there was no notable presence of salt adducts (that may create noise and “false mutation” peaks)^37^ in the preliminary manual analysis of our samples, and all other positive cases were confirmed by Sanger sequencing, which minimizes the possibility of hairpins. However, because we established Sanger sequencing as our confirmatory method for the results from MassARRAY assays and there was no confirmation of the mutation by direct sequencing in 2 independent runs, that sample was excluded from the analysis of the mutated cases. Overall, considering all the assays that were successfully performed, our samples had a very high specificity, similar to other series using this technology.^38^–^41^

Our specifically designed panel has further validated this technique as a fast and reliable alternative for testing multiple samples in a clinical basis. MassARRAY spectrometry is a high-throughput platform that maximizes the amount of data extracted from mutation profiling. The advantages include the standardized assay conditions and the homogeneous reaction format, which simplifies and minimizes the reagents and the processing time. Sanger sequencing requires amplification of multiple fragments per sample, with optimization of multiple primers and reactions, which besides time and costs, uses a greater quantity of DNA. In addition, MassARRAY technology has been demonstrated to be more sensitive than Sanger sequencing, having detection limits that range from 2.5% to 10%, depending on the specific mutation tested.^35^–^36^ In the present study, mutations involving *EZH2* and *CD79B* were chosen due to their potential role as therapeutic targets, but an increasing number of assays for various genes can be added to this platform. In the commercially available OncoCarta Panel (Sequenom, San Diego, Calif), for example, 238 mutations from 19 oncogenes can be simultaneously interrogated, and it has already been validated using limited samples, with satisfactory results.^21^

In summary, mutational status may vary in samples from the same case along the course of the disease, which corroborates a concept currently gaining acceptance that patients should be treated according to their current molecular findings, not on the basis of results obtained from previous specimens. The successful use of minimal and residual material from cytological samples allied to an innovative high-throughput multiplex platform for finding *EZH2* and *CD79B* mutations in patients with B-cell NHL exponentially increases the availability of specimens for clinical analysis and may lead to an increase in the number of patients eligible for clinical trials. Future larger studies using different molecular platforms to examine the pathogenesis of tumor progression may clarify the way in which mutations evolve over time and our understanding of the related molecular events.

## FUNDING SOURCES

No specific funding was disclosed.

## CONFLICT OF INTEREST DISCLOSURE

Dr. Saieg was a research fellow supported by the Terry Fox Foundation Strategic Health Research Training Program in Cancer Research at the Canadian Institutes of Health Research (grant TGT-53912).
